# Laparoscopic resection of a primary retroperitoneal mucinous cystadenoma: a case report

**DOI:** 10.1097/RC9.0000000000000633

**Published:** 2026-07-02

**Authors:** Omaira Rodríguez, Cristina Inchausti, Francis Guevara, Yrma Linares, Luz Galvis, Arantza Rodriguez

**Affiliations:** a Department of Surgery, Hospital de Clínicas Caracas, Caracas, Venezuela; b Faculty of Medicine, Central University of Venezuela, Caracas, Venezuela

**Keywords:** fluorescence-guided surgery, indocyanine green, laparoscopy, primary retroperitoneal mucinous cystadenoma, retroperitoneal cyst

## Abstract

**Introduction:**

Primary retroperitoneal mucinous cystadenoma (PRMC) is a rare tumor of uncertain origin, predominantly affecting women. Most cases are asymptomatic or present with nonspecific symptoms, making diagnosis challenging due to its retroperitoneal location and lack of distinct clinical signs.

**Case presentation:**

A 47-year-old woman presented with progressive abdominal distension, altered bowel habits, and flatulence. A 12-cm palpable mass was detected in the right flank. Abdominal computed tomography demonstrated a well-defined cystic lesion adjacent to the ascending colon. The patient was evaluated and treated at a tertiary care academic hospital. Elective laparoscopic excision was performed using indocyanine green for real-time intraoperative ureteral visualization. The procedure was completed without complications. Histopathological analysis confirmed PRMC, with mucinous epithelium lacking atypia and no evidence of malignancy.

**Discussion:**

Although PRMC shares features with ovarian mucinous tumors, it arises independently in the retroperitoneum and is often difficult to diagnose preoperatively due to the absence of specific clinical or radiological signs. Imaging studies can help delineate the lesion, but definitive diagnosis depends on histopathological confirmation. Complete surgical excision without rupture is essential to avoid recurrence or potential malignant transformation. Minimally invasive resection, when feasible, offers a safe and effective therapeutic option with low morbidity.

**Conclusion:**

PRMC should be considered in the differential diagnosis of retroperitoneal cystic lesions in women. Laparoscopic excision was safe and effective in this case. A long-term follow-up is recommended due to the risk of recurrence.

## Introduction

Retroperitoneal tumors are rare and complex clinical entities, accounting for less than 0.2% of all neoplasms. Among them, primary retroperitoneal mucinous cystadenomas (PRMCs) are exceptionally uncommon and pose significant diagnostic and therapeutic challenges. These tumors can be classified as benign, borderline, or malignant cystadenocarcinomas, and they typically present with nonspecific symptoms, making diagnosis difficult^[^1,2^]^.

PRMCs arise from retroperitoneal structures, with secondary lesions, metastases, and lymphadenopathies excluded from this classification. While mucinous cystadenomas are more commonly found in ovarian tissue, their occurrence in the retroperitoneum is extremely rare and primarily affects women. PRMC was first described by Handfield-Jones in 1924, and fewer than 100 cases have been reported worldwide^[^1,3,4^]^.

HIGHLIGHTS

- Primary retroperitoneal mucinous cystadenoma (PRMC) is a rare tumor, often asymptomatic and difficult to diagnose preoperatively.
- Imaging revealed a large cystic mass adjacent to the right colon, with no clear organ of origin.
- Laparoscopic excision was successfully performed, and histopathology confirmed PRMC.
- PRMC should be included in the differential diagnosis of retroperitoneal cystic lesions in women.
- Minimally invasive surgery is feasible and safe for the complete resection of such tumors.

The histogenesis of PRMC remains controversial due to the absence of epithelial tissue in the retroperitoneum. Several theories propose that these tumors may arise from ectopic ovarian tissue, teratomas, or mucinous metaplasia of mesothelial cells during embryonic development^[^5,6^]^.

Diagnosing PRMC is challenging, as most patients are asymptomatic or present with vague abdominal symptoms. Imaging may suggest the presence of a cystic lesion but is rarely conclusive; therefore, histopathological evaluation is essential for a definitive diagnosis. Surgical resection is critical not only for accurate diagnosis but also for effective treatment[7].

This case report presents a rare instance of PRMC, with emphasis on its clinical features, diagnostic approach, and surgical management. It highlights the importance of considering PRMC in the differential diagnosis of retroperitoneal cystic lesions and the key role of surgery in both diagnosis and treatment.

This case has been reported in line with the SCARE 2025 criteria[8].

## Case presentation

### Patient information

A 47-year-old woman with no significant medical history presented with a 1-year history of intermittent abdominal distension, altered bowel habits, and flatulence. The patient was evaluated and treated at a tertiary care academic hospital.

On physical examination, the patient appeared to be in stable condition. The abdomen was soft, compressible, and non-tender to palpation. A mass measuring approximately 12 cm was palpated in the right flank, extending toward the right iliac fossa. The mass was adherent to deeper planes but remained non-tender. The remainder of the physical examination was within normal limits.

### Diagnostic studies

**Figure 1.**
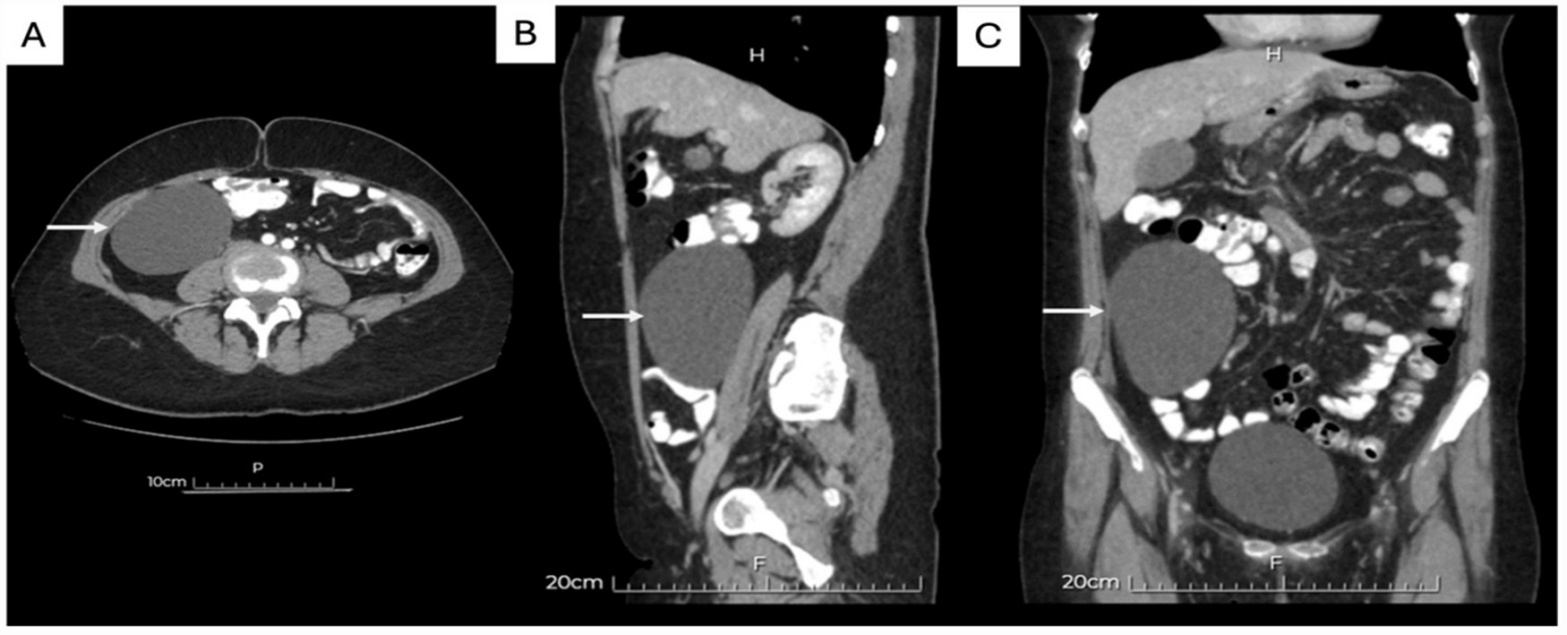
CT imaging of the retroperitoneal mass. Abdominal computed tomography in the (A) axial, (B) sagittal, and (C) coronal planes shows a large retroperitoneal cystic mass (arrows) with regular borders and no intracystic structures.

**Abdominal computed tomography (CT) scan**: Revealed a thin-walled cystic lesion in the right flank, lateral to the ascending colon, with a probable diagnosis of retroperitoneal cystic lymphangioma measuring 11.9 × 8.4 × 7.4 cm (Fig. 1).

As part of the preoperative evaluation, serum tumor markers, including carcinoembryonic antigen (CEA) and CA 19-9, were assessed and found to be within normal limits.

**Colonoscopy**: Unremarkable; no pathological findings were observed.

### Treatment performed

The patient was placed in the French position under general anesthesia. Prior to laparoscopy, a right ureteral catheter was placed cystoscopically to facilitate intraoperative ureteral identification. Pneumoperitoneum was established at 15 mmHg through a 10-mm umbilical port, followed by the placement of two 5-mm working ports in the left and right lower quadrants.

At the beginning of the procedure, 2.5 mg of indocyanine green (ICG) was administered through the ureteral catheter, resulting in immediate fluorescence visualization of the right ureter using the Karl Storz RUBINA® imaging system, which allowed continuous ureteral identification throughout the operation.

**Figure 2.**
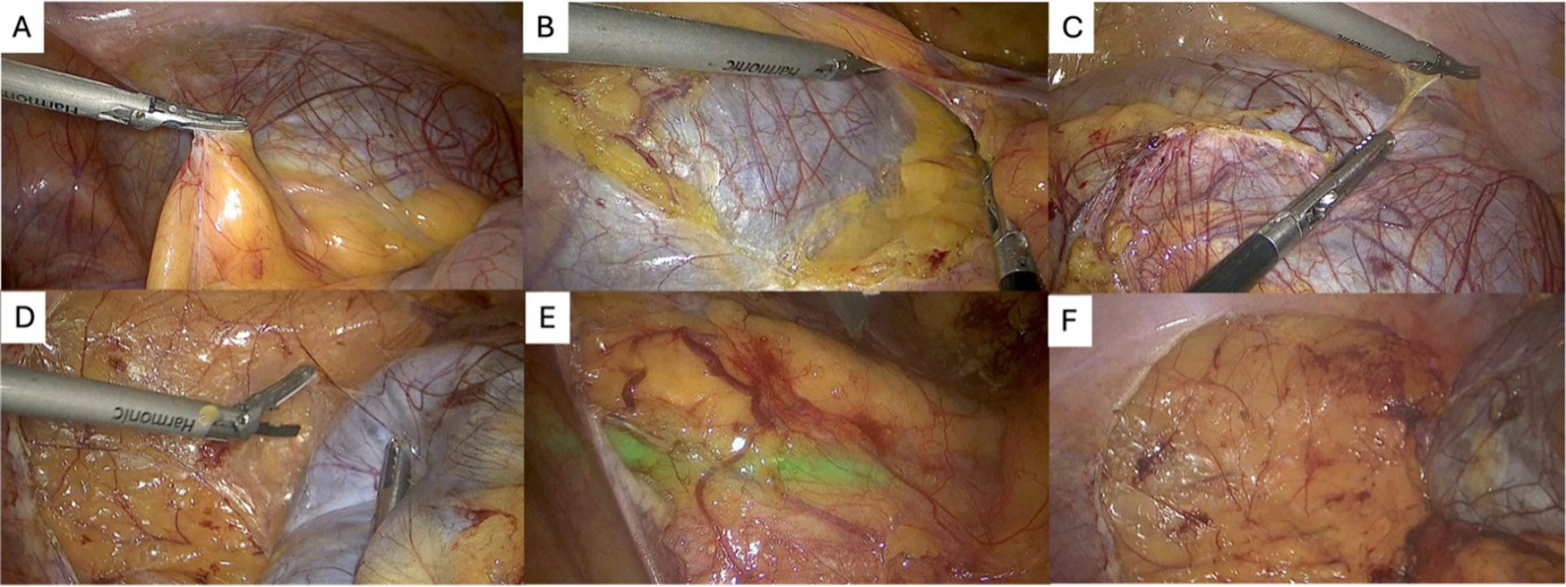
Laparoscopic excision of a retroperitoneal cyst. (A) Well-defined 12 × 8 cm cystic lesion in the right paracolic gutter, adjacent to the ascending colon. (B) Dissecting the cystic lesion from the ascending colon. (C) Carefully dissecting the lesion from the parietal wall. (D) Separating the lesion from surrounding structures. (E) Indocyanine green dye used for real-time visualization of the right ureter. (F) Final view of the resected cystic lesion.

A lateral-to-medial dissection was then initiated by incising the right white line of Toldt, allowing medial mobilization of the ascending colon and exposure of the retroperitoneal space (Fig. 2B–C). This maneuver provided clear visualization of a well-defined cystic lesion measuring approximately 12 × 8 cm in the right paracolic gutter adjacent to the ascending colon (Fig. 2A). The lesion was meticulously resected using a harmonic scalpel without injury to adjacent structures (Fig. 2D–F), while continuous fluorescence guidance facilitated preservation of the right ureter (Fig. 2E). The specimen was placed in an extraction bag, decompressed by aspiration, and removed through a small umbilical incision.

The procedure lasted 40 minutes, with no intraoperative or postoperative complications. The patient was discharged 24 hours later in stable condition and resumed normal activities within 2 weeks.

### Histological findings

**Figure 3.**
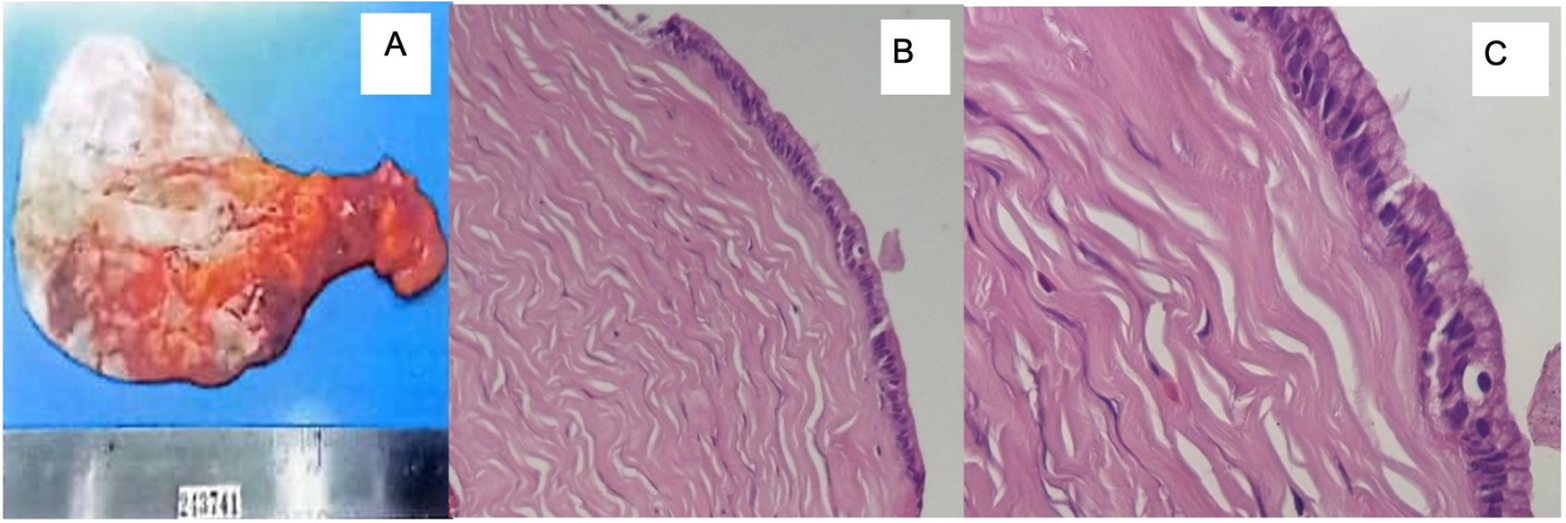
Pathological findings of the resected cyst. (A) Gross specimen showing the cyst wall. (B) Hematoxylin and eosin staining (100×) depicting fibrous tissue and vascular congestion with a single-lined epithelium. (C) Hematoxylin and eosin staining (400×) displaying columnar cells with clear cytoplasm and basal nuclei, without atypia.

Histopathological analysis confirmed the diagnosis of PRMC. The cyst was lined with columnar epithelium with clear cytoplasm and basal nuclei, consistent with a mucinous phenotype. Some areas also showed low cuboidal epithelium, with no cytologic atypia in either cell type. The cyst wall was fibrous, with zones of hyalinization and vascular congestion. No evidence of malignancy was found. The surrounding tissue consisted of adipose tissue with vascular congestion (Fig. 3).

At the 1-year follow-up, the patient remained asymptomatic, and abdominal ultrasound showed no evidence of recurrence. The patient expressed satisfaction with the procedure and recovery process, reporting a complete resolution of symptoms and a successful return to all daily activities without limitations.

## Discussion

Primary retroperitoneal mucinous tumors are rare, with PRMC being the most common subtype. These tumors share histological features with ovarian mucinous cystadenomas but can arise anywhere within the retroperitoneum, independent of the ovaries. Their histogenesis remains unclear due to the absence of native mucinous epithelium in the retroperitoneum, prompting several hypotheses. These include differentiation from ectopic ovarian tissue, teratomatous components, or mesothelial cell metaplasia induced by chronic inflammation or hormonal stimulation^[^4,9^]^.

Histologically, PRMCs are lined by mucin-secreting columnar epithelial cells, sometimes accompanied by mesothelial-like cells expressing CK5/6 and calretinin. In some cases, the presence of ovarian-type stroma has reinforced the Müllerian origin theory; however, the occurrence of PRMCs in male patients challenges this notion and supports the possibility of pluripotent mesothelial differentiation[10].

PRMCs occur almost exclusively in women, likely due to the presence of estrogen receptors in the tumor stroma. Nevertheless, a few cases have been reported in men^[^1,5^]^.

Most patients remain asymptomatic. However, similar to other retroperitoneal masses, PRMCs may produce nonspecific symptoms when they become large enough to compress or displace adjacent organs. The most commonly reported symptoms include abdominal discomfort, palpable mass, bloating, flatulence, or a sensation of fullness, as observed in our case^[^2,4^]^.

Imaging modalities such as CT and magnetic resonance imaging often reveal a unilocular, homogeneous cystic lesion within the retroperitoneum, which can raise suspicion for PRMC. However, imaging alone is not diagnostic. CT remains the gold standard for assessing size, wall thickness, location, calcifications, internal architecture, enhancement patterns, cyst margins, and potential invasion of adjacent organs or peritoneal structures^[^4,9,11,12^]^.

Percutaneous biopsy techniques, including fine-needle aspiration, remain controversial due to their limited diagnostic yield and potential risk of tumor spillage^[^2,7^]^.

Tumor markers such as CEA and CA 19-9 have shown poor sensitivity and specificity in PRMC, though isolated cases with elevated levels have been reported[1].

The treatment of choice for PRMC is complete surgical excision, ensuring the capsule remains intact to minimize the risk of infection, recurrence, or potential malignant transformation. Although PRMCs are generally considered benign or of low malignant potential, complete resection is critical. Laparoscopic resection has gained favor due to its minimally invasive nature, faster recovery, and proven safety^[^2,12^]^. However, successful laparoscopic resection requires careful patient selection and meticulous technique. Ideal candidates include those with well-encapsulated lesions without signs of invasion or dense adhesions. Key technical considerations include maintaining adequate pneumoperitoneum, preventing cyst rupture to avoid potential peritoneal contamination, and careful dissection to preserve surrounding structures, particularly the ureters. In our case, operative time was 40 minutes, which was shorter than operative times reported in some laparoscopic retroperitoneal resections^[^13,14^]^. This may reflect favorable lesion characteristics, clear anatomical delineation, and surgeon experience.

The use of ICG for intraoperative ureteral visualization represents a valuable adjunct in retroperitoneal surgery and enhances procedural safety. Most previously reported PRMC cases have focused on complete surgical excision, whereas adjunctive fluorescence-guided ureteral identification has been rarely described in this specific setting. Although ICG-guided ureteral identification is well established in colorectal and gynecologic surgery, its application in PRMC resection remains uncommon^[^15–17^].^ In our case, ICG administration through a ureteral catheter enabled real-time fluorescence guidance, facilitating safe retroperitoneal dissection and continuous monitoring of ureteral integrity throughout the procedure. This technique may be particularly useful in cases where anatomic landmarks are limited or when lesions are in close proximity to vital structures, potentially reducing the risk of ureteral injury^[^18,19^]^.

The differential diagnosis of retroperitoneal cystic lesions is broad and includes both benign and malignant conditions, such as retroperitoneal lymphangioma, mesothelioma, teratoma, Müllerian cyst, lymphocele, and urinoma. Comprehensive clinical evaluation and imaging are essential to distinguish PRMC from these entities[7].

Several limitations of this case report should be acknowledged. First, although follow-up ultrasonography at 1 year demonstrated no evidence of recurrence, this surveillance period remains relatively short for a comprehensive long-term assessment. Second, as a single case report, the generalizability of our experience with ICG-guided dissection and our favorable operative time requires validation in larger series. Finally, the optimal imaging surveillance protocol and duration for patients following PRMC resection have not been standardized in the literature.

In conclusion, PRMC is a rare but relevant entity that should be considered in the differential diagnosis of retroperitoneal cystic lesions, particularly in women. Given its nonspecific clinical presentation, accurate diagnosis relies on histopathological confirmation. When technically feasible, laparoscopic excision offers a safe and minimally invasive approach, as supported by existing literature and our case. The adjunctive use of ICG for ureteral visualization may enhance the safety of retroperitoneal dissection and warrants further investigation. Follow-up imaging at 1 year confirmed complete excision with no evidence of recurrence. Although optimal surveillance protocols for PRMC remain undefined, outcomes after complete resection are generally favorable, with long-term recurrence data remaining limited because of the rarity of this entity.

## Data Availability

All data relevant to the case are included in the article.
